# A Revised Australian Dietary Guideline Index and Its Association with Key Sociodemographic Factors, Health Behaviors and Body Mass Index in Peri-Retirement Aged Adults

**DOI:** 10.3390/nu8030160

**Published:** 2016-03-11

**Authors:** Maree G. Thorpe, Catherine M. Milte, David Crawford, Sarah A. McNaughton

**Affiliations:** Centre for Physical Activity and Nutrition Research, School of Exercise and Nutrition Sciences, Deakin University, 221 Burwood Hwy, Burwood, Victoria 3125, Australia; catherine.milte@deakin.edu.au (C.M.M.); david.crawford@deakin.edu.au (D.C.); sarah.mcnaughton@deakin.edu.au (S.A.M.)

**Keywords:** diet quality, diet quality index, dietary guidelines, Australian, older adults, BMI

## Abstract

The Dietary Guideline Index, a measure of diet quality, was updated to reflect the 2013 Australian Dietary Guidelines. This paper describes the revision of the index (DGI-2013) and examines its use in older adults. The DGI-2013 consists of 13 components reflecting food-based daily intake recommendations of the Australian Dietary Guidelines. In this cross-sectional study, the DGI-2013 score was calculated using dietary data collected via an 111-item food frequency questionnaire and additional food-related behaviour questions. The DGI-2013 score was examined in Australian adults (aged 55–65 years; *n* = 1667 men; 1801 women) according to sociodemographics, health-related behaviours and BMI. Women scored higher than men on the total DGI-2013 and all components except for dairy. Those who were from a rural area (men only), working full-time (men only), with lower education, smoked, did not meet physical activity guidelines, and who had a higher BMI, scored lower on the DGI-2013, highlighting a group of older adults at risk of poor health. The DGI-2013 is a tool for assessing compliance with the Australian Dietary Guidelines. We demonstrated associations between diet quality and a range of participant characteristics, consistent with previous literature. This suggests that the DGI-2013 continues to demonstrate convergent validity, consistent with the original Dietary Guideline Index.

## 1. Introduction

The Dietary Guideline Index (DGI) [1] is a comprehensive food-based diet quality index that reflects adherence to the Dietary Guidelines for Australian Adults [2,3]. It has been shown to have an inverse relationship with daily fat and energy consumption and a positive relationship with dietary fiber and important micronutrients such as vitamin C, folate, calcium, and iron [1]. A higher Dietary Guideline Index score has been associated with reduced risk of Type 2 diabetes, hypertension and obesity [4,5], and people with a higher score have demonstrated less weight gain over 15 years compared to those with a lower score [6]. In 2013, the Australian National Health and Medical Research Council released revised Australian Dietary Guidelines (ADG) [7] based on an updated review of the scientific evidence [8]. A revision of the Dietary Guideline Index is necessary in order to capture key changes to the guidelines in this measure of diet quality.

Increased longevity combined with the ageing of the baby boomers is leading to growth in the proportion of older people worldwide [9]. Older adults are an understudied population in nutrition, however, research suggests that poor diet quality among this population is associated with reduced quality of life [10,11], increased cardiometabolic risk [4,12], and increased risk of mortality [13]. It is important that older adults maintain good health and quality of life to continue their active contribution to society and to manage the economic pressures of growing health and aged care service demand [9]. An estimated 80% of health problems associated with old age could be prevented or postponed through lifestyle change and interventions within 50+ age groups are likely to provide benefits [14]. Peri-retirement is a transitional period of life where adults may experience changes in lifestyle, including changes in family dynamics and employment. These events have potential to influence behaviours such as diet [15]. Exploring diet quality in peri-retirement aged adults (defined as 55–65 years) will help identify adults at risk of poor diet and will also help shape preventative campaigns that target at risk groups during a time of life in which lifestyle changes are likely to occur.

The aims of this paper are to revise the Dietary Guideline Index (DGI-2013) and to examine the diet quality of peri-retirement aged adults by examining the DGI-2013’s associations with key sociodemographic characteristics, health-related behaviours and body mass index (BMI).

## 2. Materials and Methods

### 2.1. Participants and Recruitment

The data was sourced from the Wellbeing Eating and Exercise for Long Life (WELL) study, a cohort study of Australian adults aged 55–65 years [16]. This study examined nutrition and physical activity as well as exploring determinants of these behaviours in a longitudinal study. The current study utilizes the baseline WELL data. A random sample of 11,256 Australian adults aged 55–65 years was selected from the Australian Electoral Commission’s electoral roll in October 2009 and invited to participate in the WELL study, described in detail elsewhere [16]. Briefly, a sample of men and women were selected from rural and urban Victorian postcodes across three tertiles of socioeconomic position [17]. Of the 11,256 invitations sent, 475 could not be delivered or participants did not meet the studies age criteria, resulting in 10,781 eligible participants. A total of 4082 adults completed a survey in 2010 (38% response rate).

### 2.2. Ethics

The Deakin University Human Research Ethics Committee approved the WELL study protocol (approval No. 2009-105) and all participants provided informed consent, as a requirement to obtain approval.

### 2.3. Dietary Intake

Dietary intake was assessed using a 111-item Food Frequency Questionnaire (FFQ). The FFQ was developed for and used in the 1995 Australian National Nutrition Survey [18], has been used in other Australian cohort studies and updated based on the current food supply [19,20]. The FFQ assessed participant’s dietary intake over the previous six months, with nine response categories for each item ranging from “never or less than once a month” to “6+ times per day”. Additional food-based behaviours were assessed via previously developed questionnaire tools including daily fruit and vegetable intake, salt use, trimming the fat from meat, type of bread and type of milk usually consumed [18,21,22,23,24,25]. Poorly completed FFQs were excluded from the study (missing greater than 10% of data [26]). For the remaining participants, missing responses were coded as not consumed [18].

### 2.4. Sociodemographic Factors and Health Behaviors

Self-reported sociodemographic data including age, place of birth, employment status and education as well as health-related behaviours were collected. Participants reported their current smoking status (never, smoked, former smoker or current smoker) and physical activity in the seven days prior to the survey was assessed using the long version of the International Physical Activity Questionnaire (IPAQ) [27]. IPAQ collects data on the frequency, intensity, and duration of leisure time physical activity. Minutes of activity per week were calculated by summing the minutes of moderate intensity physical activity and twice the number of minutes spent participating in vigorous intensity physical activity [27]. A variable to indicate if participants were meeting the physical activity recommendations of at least 150 min of activity per week was created [28].

### 2.5. Body Mass Index

BMI was calculated using self-reported height and weight and categorized according to the World Health Organization criteria (Healthy: BMI ≥ 18.5 to < 25 kg/m^2^; Overweight: BMI ≥ 25 to < 30 kg/m^2^; Obese: BMI ≥ 30 kg/m^2^) [29].

### 2.6. Revision of DGI-2013

The DGI-2013 was developed to reflect compliance with the 2013 Australian Dietary Guidelines. It is based on the previous Dietary Guideline Index [1] and was updated with major changes relating to age and sex-based food intake recommendations, changes in terminology, and inclusion of a new component relating to unsaturated fats [7]. It was developed for adults and guidelines relating to breastfeeding and food hygiene were not included, as these are not relevant to adult’s dietary intake.

The DGI-2013 is comprised of 13 components (Table 1). Each component is scored out of ten, with zero indicating the guideline was not met and ten indicating the guideline was sufficiently met. The total score ranged from 0 to 130; a higher score indicating greater compliance with dietary guidelines and hence, higher diet quality. The components are separated into two categories (1) those that reflect adequate intake of nutritious foods and (2) those that reflect moderation or limited intake of foods and drink high in saturated fat and/or added sugar, added salt or alcohol and low in fiber, known as discretionary foods. Several components have sub-components in order to capture important food choices described in the ADG [7], for example, choosing mostly wholegrain or high fiber cereals, and choosing lean meat. The cut-offs used to obtain the maximum score for components were guided by the age and sex-specific food-based daily recommendations outlined in the ADG [7]. For components that assess adequate intake of nutritious foods, a maximum score is awarded if the daily consumption meets or exceeded the recommendations outlined in the ADG [7] and a proportionate score was given for those who fall below this, a recommended practice for scoring dietary indices [30,31]. For components that reflect guidelines for limiting intake, a score of ten was awarded for remaining below the cut-off or zero for exceeding it.

The DGI-2013 contains indicators that reflect adequate intake of nutritious foods from core food groups (vegetable, fruit (not including juice), cereal, dairy and alternatives and meat and alternatives) as well as the food variety within these core groups [1,7]. These are retained from the original index with amendments to the scoring criteria according to the revised recommendations. The food variety component was based on the variety of foods consumed within the core food groups using the same method outlined in the original index [1] and analogous to the Recommended Food Score [13].

The DGI-2013 also reflect recommendations for moderation or limited intake of discretionary foods. “Discretionary foods” describe energy-dense food and drink that are not essential to nutrition [7] and were known as “extra foods” in the previous dietary guidelines. Revisions to the scoring cut-off values were based on the revised food intakes recommendations [7].

A new component, moderate unsaturated fat is included in the DGI-2013 to reflect the new guidelines that makes allowances for moderate intake of poly- and mono-unsaturated fat intake. There is good evidence to suggest that replacing dietary saturated fatty acids with unsaturated fatty acids is associated with improved blood lipid profiles and reduced inflammation markers [7].

### 2.7. Application of the Revised Dietary Guideline Index in the WELL Study

The DGI-2013 was applied to the WELL study sample using the food-based recommendations specific for 51–70 years old to determine cut-off values. The total daily intake of food groups was calculated by summing the daily intake frequency of the food or beverage items within the group. To avoid over-estimation of fruit and vegetable intake based on the FFQ responses [34], daily intake was based on two short behaviour questions “about how many serves of fruit/vegetable do you usually eat per day?”. Response options for vegetable intake in the questionnaire did not allow estimation of the 2013 dietary recommendation for men (5.5 servings); therefore, the cut-off for a maximum total vegetable score in men was rounded down to at least five servings per day, consistent with the previous recommendations and DGI.

The level of wholegrain or high fiber cereal consumption was determined by the behavioural question “what type of bread do you usually eat?” Scores were given according to the amount of fiber typically in the bread type reported, with the following options; I don’t eat bread, high fiber white bread, white bread, wholemeal bread, rye bread, multigrain bread and other bread.

### 2.8. Statistical Analysis

Of the 4082 participants, 1667 men and 1801 women aged 55–65 years old had complete data required to calculate the DGI-2013 as well as complete participant characteristics used for this analysis. Mean total DGI-2013 score and mean scores for each component were calculated and compared between gender using *t*-tests. The percentage meeting the recommendations were calculated and compared between gender using chi-square. Associations between the DGI-2013 score and participant sociodemographic variables, health-related behaviours and BMI were investigated using chi-square for categorical data and analysis of variance for continuous data, stratified by sex.

## 3. Results

The distributions of participant characteristics are shown in Table 2. The mean age for men and women was the same (mean age ± standard deviation (SD); 59.9 ± 3.1 years). More men than women were working full-time and had achieved higher levels of education, men were more likely to be current or former smokers, less likely to be meeting the physical activity guidelines and to be overweight or obese than women.

Women scored higher than men on the overall DGI-2013 (mean DGI-2013 score ± standard error (SE) 89.7 ± 0.3 *vs.* 81.5 ± 0.4, respectively; *p* < 0.001) (Table 3) and on all the components except for the dairy component (Table 3). Men and women scored the lowest on the limit discretionary food component (reflecting poorer compliance) with a mean component score ± SE of 2.6 ± 0.11 and 3.8 ± 0.11, respectively out of the total component score of 10. This was closely followed by the food variety component (4.0 ± 0.03 and 4.6 ± 0.03 points) and daily vegetable intake (4.4 ± 0.06 and 5.8 ± 0.06 points). Men and women scored highest on moderating unsaturated-fat (9.8 ± 0.03 and 8.6 ± 0.08 points) and alcohol intake (8.3 ± 0.09 and 9.4 ± 0.06 points). Few participants were meeting the guidelines for fruit and vegetable intake, with only 6% of men and 13% of female meeting the daily vegetable recommendations and 52% of men and 69% of women meeting the daily fruit recommendations. No participants achieved a full score for food variety.

The results indicated that those with a lower DGI-2013 were more likely to be from rural areas (men only), working full-time (men only), have achieved a lower level of education, smoked, not meeting the physical activity guidelines and have a higher BMI (Table 4).

## 4. Discussion

The DGI-2013 is a revised tool that can be used for assessing diet quality of Australian adults based on the Australian Dietary Guidelines. In a sample of peri-retirement aged Australians, a higher diet quality was associated with being female, higher education, not smoking, meeting physical activity guidelines and a lower BMI. These results are consistent with previous studies [1,4,13]. Among the peri-retirement aged adults, overall compliance with the dietary guidelines was poor. Both men and women showed the least compliance with guidelines for limiting discretionary food, followed by the food variety and vegetable components, and they showed greatest compliance with moderating unsaturated-fat and alcohol intake.

Our results suggest that men from urban areas had higher diet quality than those from rural areas. Perhaps men are influenced by rural-related disadvantage such as access and availability of nutritious food [35], while women, tending to be more health conscious [36], are more likely to overcome these barriers. It is interesting that men who worked part-time or less had higher diet quality than those working full-time. Lack of time and competing priorities is perceived as a barrier to healthy eating [37], and, therefore, those who work may have less time to focus on healthy eating. However, income is also a determinant of diet quality [38], and may be important in peri-retirement adults who experience a drop in income as they change work-loads or retire [39]. Little is known about the economic determinants of diet among older adults [40]. There may be complex interactions between diet and socioeconomic exposures, particularly during a life-stage transition such as peri-retirement.

We found a higher diet quality was associated with lower BMI. This is consistent with previous cross-sectional [41] and longitudinal studies [6,42,43]. Furthermore, the DGI-2013 has been positively associated with health-related quality of life at two-years in the WELL study sample [11]. This demonstrates convergent validity consistent with the original Dietary Guideline Index, with adherence to the DGI-2013 protecting against negative health-outcomes, a goal of the ADG [7]. However, further work, particularly in longitudinal analyses, is required to confirm these findings.

Another key finding of this study is that only 31% of men and 53% of women were meeting the daily intake recommendation for lean meat and alternatives, a protein-rich food group. This is substantially lower than earlier results reported from an Australian adult population (19 years old) in which 87%–90% of men and women met the previous guideline [1]. This is concerning for this age group given protein is important to help maintain muscle mass [44]. Low compliance rates were also observed for the fruit, vegetable and cereal guidelines, consistent with other studies [1,45].

There are a number of considerations to address when developing and using diet quality indices, including decisions regarding the components or indicators for inclusion in the index [30]. Previous diet quality indices have incorporated fish consumption as an isolated component of their score due to evidence to support the health benefits of fish [30]; however, this is not included in the DGI-2013 [46]. The components in the DGI-2013 were guided by the ADG [7] which do not include daily intake recommendations for fish. Fish serves are counted towards the serves of lean meat and alternatives. Considerations were made regarding whether the DGI-2013 should include a component reflecting recommendation to substitute poly- and mono- unsaturated fatty acids for saturated fatty acids. Previous dietary indices have included a ratio of fat types [30]. It is unclear if there are equivalent health effects for poly- *vs.* mono- unsaturated fatty acid consumption and similarly for omega-3 fatty acid *vs.* omega-6 [47], and perhaps a more complex indicator would be required [30]. In addition, the DGI is designed as a food-based score, people consume whole foods rather than individual nutrients and therefore nutrient-based indicators were not included. Instead, the moderate unsaturated fat component was included inline with the ADG recommendation to include a small allowance of unsaturated oils, fats and spreads. Consideration of alcohol consumption has previously been highlighted as contentious with a suggestion that non-consumers should be disadvantaged in the diet quality scoring system, due to the evidence of positive effects of moderate intake [30,48,49]. However, these findings have been challenged and the ADGs maintain that abstainers have better health outcomes than heavy drinkers [7].

This study has a number of strengths and limitations. Firstly, the results were limited by the modest response rate (38%); however, the participant characteristics were comparable to national population statistics. For example, the WELL study participants had similar levels of employment in comparison to national figures (60% *vs.* 61% in full time or part time employment) and they were marginally more highly educated (28% *vs.* 19% completing a university degree or higher). Participants were less likely to be overweight or obese in comparison to national data (64% *vs.* 74%) and were less likely to be current smokers (12% *vs.* 15%) [45,50,51]. Similar proportions of the WELL sample were meeting fruit (10% *vs.* 11%) and vegetable (61% *vs.* 56%) recommendations compared to the national population of the same age [45].

The cross-sectional nature limits this study, as a causal relationship cannot be assumed. The applications of the DGI-2013 in the WELL study was also limited by the available dietary intake data, in particular, there was no information collected on portion sizes to compliment the FFQ data; however, this is consistent with previous applications of the DGI [1]. There were few foods that could be used as indicators of unsaturated fat intake; and participants following a vegetarian diet were not identifiable and, therefore, they may have been disadvantaged in the score calculation. However, the 2011–2012 Australian Health Survey found that only 2% of 51 to 70 years old avoid meat [45] minimising implications on our findings.

The use of the food-based index to assess diet quality is a significant strength as diet research has shifted from exploring individual nutrients to whole foods and food patterns, to account for the interactions of the nutrients and non-nutrient components of foods *in vivo* [30]. Another strength was the use of a diet quality tool that has previously been shown to reflect intake of key nutrients and discriminate across varying socioeconomic factors, health behaviours and health outcomes [1,4,5,6]. This study updated this tool so that it reflects the best scientific evidence available [7,8]. Exploring this understudied population of peri-retirement aged adults is a significant aspect of this work as it helps improve our understanding of diet within the context of a unique life stage.

## 5. Conclusions

Adults aged 55–65 years demonstrated poor diet quality according to the DGI-2013. Those at risk of the poorest diet quality were those who reported lower education, negative health behaviours including smoking and low physical activity, and a higher BMI, highlighting a group of peri-retirement aged adults at risk of poor health. This study demonstrated expected associations between diet quality and a range of participant characteristics including BMI consistent with previous literature. This suggests that the DGI-2013 continues to demonstrate convergent validity, consistent with the original DGI. Further investigation of the DGI-2013 and other health outcomes are required to confirm these findings, particularly in longitudinal studies.

## Figures and Tables

**Table 1:** Components and scoring methods of the revised Dietary Guideline Index (DGI-2013).

Dietary Guideline	Indicator and Description	Criteria for Maximum Score ^1^	Criteria for Minimum Score	Maximum Score
Guidelines for adequate intake				
1. Enjoy a wide variety of nutritious foods	Food variety ^2^: proportion of food from each of the 5 core food groups eaten at least one serve per week	100%	0%	10
2. Plenty of vegetables	Total vegetable intake: servings of vegetables per day	19–50 y: M ≥ 6, F ≥ 5	0	10
		51–70 y: M ≥ 5.5, F ≥ 5		
		> 70 y: M ≥ 5, F ≥ 5		
3. Fruit	Total fruit intake: servings of fruit per day	≥2	0	10
4. Grain (cereal) foods	Total cereal intake: servings of grains per day	19–50 y: M ≥ 6, F ≥ 6	0	5
		51–70 y: M ≥ 6, F ≥ 4		
		>70 y: M ≥ 4.5, F ≥ 3		
	Mostly wholegrain or high fiber cereals: Type of bread usually consumed	Wholemeal bread	White bread	5
5. Lean meat and poultry, fish, eggs, nuts and seeds, and legumes/beans	Total meat and alternative: servings per day	19–50 y: M ≥ 3, F ≥ 2.5	0	5
		51–70 y: M ≥ 2.5, F ≥ 2		
		>70 y: M ≥ 2.5, F ≥ 2		
	Lean meat: proportion of lean meats and alternatives to total meat and alternatives per day	100%	0%	5
6. Milk, yoghurt, cheese and/or their alternatives ^3^	Total dairy and alternative: servings per day	19–50 y: M ≥ 2.5, F ≥ 2.5	0	10
		51–70 y: M ≥ 2.5, F ≥ 4		
		>70 y: M ≥ 3.5, F ≥ 4		
7. Drink plenty of water	Total beverage intake ^4^: servings per day	M ≥ 10; F ≥ 8	0	5
	Water ^5^: proportion of water to total beverage intake per day	≥50%	0%	5
Guidelines to limit or moderate intake				
8. Limit intake of foods containing saturated fat, added salt, added sugars and alcohol	Limit discretionary foods	M ≤ 3; F ≤ 2.5	M > 3; F > 2.5	10
9. Limit intake of foods high in saturated fat	Trim meat: trimming fat from meat	Usually	Never or rarely	5
	Choose reduced-fat milk: type of milk usually consumed	Skim, low or reduced fat milk	Whole milk	5
10. Small allowance of unsaturated oils, fats or spreads	Unsaturated spreads and oils: servings per day	19–50 y: M ≤ 4, F ≤ 2	M > 4; F > 2	10
		51–70 y: M ≤ 4, F ≤ 2		
		>70 y: M ≤ 2, F ≤ 2		
11. Limit intake of foods and drinks containing added salt	Salt use: salt added during cooking	Never or rarely	Usually	5
	Salt use: salt added during the meal	Never or rarely	Usually	5
12. Limit intake of foods and drinks containing added sugars	Limit extra sugar ^6^: servings per day	M ≤ 1.5; F ≤ 1.25	M > 1.5; F > 1.25	10
13. If you choose to drink alcohol, limit intake	Limit alcohol: servings per day	≤2	>2	10

^1^: Criteria for maximum scores were derived from the Australian Dietary Guidelines [7] unless otherwise noted; y: years; M: Male; F: Female; ^2^: Food variety was measured and scored using a similar method to the Recommended Food Score [13]; ^3^: Choosing reduced fat dairy is captured in the “Limit intake of foods high in saturated fat” component; ^4^: The Eat for Health Australian Dietary Guidelines do not have specific recommendations for beverage consumption and recommended the guidelines found in the Nutrient Reference Values for Australia and New Zealand [32]; ^5^: The proportion of water to total beverage intake was derived from US beverage guidelines [33]; ^6^: Since added sugar intake is not recommended there are no cut-off values for the number of recommended servings, instead half of the maximum discretionary food cut-off were used consistent with the original DGI [1].

**Table 2:** Sociodemographic characteristics, health-related behaviours and body mass index (BMI) of the participants of the Wellbeing Eating and Exercise for a Long Life study in 2010 ^1^.

	Men (*n* = 1667)	Women (*n* = 1801)	*p*-Value ^2^
Mean age (years ± SD)	59.9 ± 3.1	59.9 ± 3.1	0.98
Born in Australia			
Yes	78.8	81.2	0.08
No	21.2	18.8	
Urban/Rural			
Urban	47.6	47.8	0.94
Rural	52.4	52.3	
Employment status			
Working full-time	48.1	20.5	<0.001
Working-part time	18.7	32.1	
Not working	33.2	41.4	
Education			
No formal qualifications and up to year 10	31.0	39.0	<0.001
Year 12, Trade/apprenticeship or certificate/diploma	39.5	33.2	
University degree and higher	29.5	27.8	
Smoking status (%)			
Never smoked	43.8	56.5	<0.001
Former smoker	43.0	32.8	
Current smoker	13.3	10.7	
Meeting physical activity guidelines			
Yes	47.8	52.9	0.003
No	52.2	47.1	
BMI			
Healthy (BMI ≥ 18.5 to < 25 kg/m^2^)	27.6	44.0	<0.001
Overweight (BMI ≥ 25 to < 30 kg/m^2^)	47.9	32.5	
Obese (BMI ≥ 30 kg/m^2^)	24.5	23.4	

^1^: Values are percentages unless otherwise specified; ^2^: Statistical significance by sex using ANOVA or Chi-square.

**Table 3:** Mean and standard error 2013 Dietary Guideline Index component scores and percent meeting dietary guidelines.

	DGI-2013 Component Score ^1^				% Meeting Guideline ^2^	
DGI-2013 Component Scores	Men		Women		Men	Women
1. Food variety	4.0	(0.03)	4.6	(0.03) **	0	0
2. Vegetables	4.4	(0.06)	5.8	(0.06) **	6	13 **
3. Fruit	7.0	(0.08)	8.2	(0.07) **	52	69 **
4. Cereal (total)	4.5	(0.05)	5.4	(0.05) **	1	2
4a. servings per day ^3^	1.9	(0.02)	2.5	(0.03)	2	7 **
4b. mostly wholegrain ^3^	2.6	(0.04)	2.9	(0.04)	26	29 *
5. Meat and alternatives (total)	7.8	(0.04)	8.6	(0.03) **	2	7 **
5a. servings per day ^3^	3.7	(0.03)	4.2	(0.02)	31	53 **
5b. mostly lean ^3^	4.1	(0.02)	4.4	(0.01)	5	12 **
6. Dairy and alternatives	5.7	(0.07)	4.1	(0.06) **	16	4 **
7. Fluid intake	6.3	(0.06)	7.9	(0.05) **	4	21 **
8. Limit discretionary foods	2.6	(0.11)	3.8	(0.11) **	26	38 **
9. Limit saturated fat	7.3	(0.08)	8.6	(0.05) **	48	68 **
10. Moderate unsaturated-fat	9.8	(0.03)	8.6	(0.08) **	98	86 **
11. Limit added salt	6.0	(0.08)	6.6	(0.08) **	28	35 **
12. Limit extra sugar	7.8	(0.10)	8.1	(0.09) *	78	81 *
13. Limit alcohol	8.3	(0.09)	9.4	(0.06) **	83	94 **
Total DGI-2013	81.5	(0.36)	89.7	(0.32) **	-	-

^1^: Data are mean (SEM) * Different from men <0.05 ** *p* < 0.001; ^2^: Those with a maximum DGI-2013 component score were considered meeting the guideline * Different from men <0.05 ** *p* < 0.001; ^3^: The sub-components range was 0–5, while the remaining components were 0–10 and the total DGI-2013 scores range was 0–130.

**Table 4:** Mean and standard error of the 2013 Dietary Guideline Index score according to participant characteristics, Wellbeing Eating and Exercise for a Long Life study at Time 1, 2010.

	Men (*n* = 1667)			Women (*n* = 1801)		
	Mean DGI-2013	SE	*p*-Value ^1^	Mean DGI-2013	SE	*p*-Value ^1^
Born in Australia						
Yes	81.1	(0.41)	0.224	89.7	(0.36)	0.70
No	82.3	(0.77)		90.0	(0.72)	
Urban/Rural						
Urban	83.0	(0.52)	<0.001	89.7	(0.48)	0.94
Rural	80.1	(0.51)		89.7	(0.44)	
Employment status						
Working full-time	80.5	(0.51) ^a^	0.020	89.9	(0.71)	0.60
Working-part time	82.8	(0.87) ^a,b^		90.1	(0.57)	
Not working	82.3	(0.64) ^b^		89.4	(0.47)	
Education						
No formal qualifications and up to year 10	78.2	(0.65) ^a^	<0.001	87.4	(0.54) ^a^	<0.001
Year 12, Trade/apprenticeship, certificate/diploma	80.7	(0.58) ^b^		90.2	(0.56) ^b^	
University degree and higher	86.0	(0.62) ^c^		92.4	(0.55) ^c^	
Smoking status						
Never smoked	84.1	(0.49) ^a^	<0.001	90.8	(0.42) ^a^	<0.001
Former smoker	82.2	(0.55) ^b^		89.6	(0.55) ^a^	
Current smoker	70.6	(1.03) ^c^		84.2	(1.09) ^b^	
Meeting physical activity guidelines						
Yes	84.3	(0.50)	<0.001	92.5	(0.41)	<0.001
No	78.9	(0.51)		86.6	(0.49)	
BMI						
Healthy (BMI ≥ 18.5 to <25 kg/m^2^)	83.2	(0.74) ^a^	0.008	90.3	(0.49) ^a^	0.019
Overweight (BMI ≥ 25 to <30 kg/m^2^)	81.2	(0.51) ^ab^		90.1	(0.56) ^a^	
Obese (BMI ≥ 30 kg/m^2^)	80.1	(0.70) ^b^		88.1	(0.67) ^b^	

^1^: Statistical significance by participant characteristics using ANOVA or *t*-test; ^a^, ^b^, ^c^: Different letters indicate where the differences lie according to Sidak *post hoc* test.

## Abbreviations

The following abbreviations are used in this manuscript:

ADG	Australian Dietary Guidelines
BMI	Body Mass Index
DGI-2013	Revised Dietary Guidelines Index
FFQ	Food Frequency Questionnaire
IPAQ	International Physical Activity Questionnaire
SE	Standard Error
SD	Standard Deviation
WELL	Wellbeing Eating and Exercise for Long Life study
